# Development of effective model for non-destructive detection of defective kiwifruit based on graded lines

**DOI:** 10.3389/fpls.2023.1170221

**Published:** 2023-08-25

**Authors:** Feiyun Wang, Chengxu Lv, Lizhong Dong, Xilong Li, Pengfei Guo, Bo Zhao

**Affiliations:** National Key Laboratory of Agricultural Equipment Technology, Chinese Academy of Agricultural Mechanization Sciences Group Co., Ltd, Beijing, China

**Keywords:** kiwifruit, grading line, SPD-Conv, DWConv, real time, non-destructive detection

## Abstract

The accurate detection of external defects in kiwifruit is an important part of postharvest quality assessment. Previous studies have not considered the problems posed by the actual grading environment. In this study, we designed a novel approach based on improved Yolov5 to achieve real-time and efficient non-destructive detection of multiple defect categories in kiwifruit. First, a kiwifruit image acquisition device based on grading lines was developed to enhance the image acquisition. Subsequently, a kiwifruit dataset was constructed based on the external defect characteristics and a new data enhancement method was proposed to augment the kiwifruit samples. Thereafter, the SPD-Conv and DW-Conv modules were combined to improve Yolov5s, with EIOU as the loss calculation function. The results demonstrated that the improved model training loss value was 0.013 lower, the convergence was accelerated, the number of parameters was reduced, and the computational effort was increased. The detection accuracies of the samples in the test set, which included healthy, leaf-rubbing damaged, healed cuts or scarred, and sunburned samples, were 98.8%, 98.7%, 97.6%, and 95.9%, respectively, with an overall detection accuracy of 97.7%. The detection time was 8.0 ms, thereby meeting real-time sorting demands. The average detection accuracy and model size of SSD, Yolov5s, Yolov7, and Yolov5-Ours were compared. When the confidence threshold was 0.5, the detection accuracy of Yolov5-Ours was 10% and 6.4% higher than that of SSD and Yolov5s, respectively. In terms of the model size, Yolov5-Ours was approximately 6.5- and 4-fold smaller than SSD and Yolov7, respectively. Thus, Yolov5-Ours achieved the highest accuracy, adaptability, and robustness for the detection of all kiwifruit categories as well as a small volume and portability. These results can provide technical support for the non-destructive detection and grading of agricultural products in the future.

## 1 Introduction

Kiwifruit is characterized by a soft texture, sweet and sour taste, and richness in amino acids and minerals. The detection and grading of kiwifruit are key aspects of postharvest processing and provide important support for value-added commercialization (Fu et al., 2018; Li et al., 2022).

In China, the grading of kiwifruits from different cities is primarily conducted by manual sorting at present, which is inefficient and subjective. Existing sorting equipment, such as mechanical size grading and weight grading, cannot identify the external defects of the fruit. Thus, computer vision is being applied increasingly to agricultural products with the developments in image processing technology (Liu et al., 2020; Tian et al., 2021).

Traditional image processing methods usually achieve fruit recognition and detection by combining the extraction of shallow information, such as the color, size, and texture of the target, using techniques such as segmentation and discriminative models. Cui et al. (2012) proposed the use of a near-infrared light source for image acquisition and realized the extraction of scratch, decay, and sun-burning defects using segmentation. Yang et al. (2021) used the K-means clustering algorithm to segment the surface of kiwifruit and reject defective fruits according to the darker color of surface defects, such as fruit scars and disease spots, compared with those of normal fruits. Subsequent studies (Zhou et al., 2012; Liu and Gai, 2020) used an image segmentation algorithm to extract the contours of the fruit in an image to meet the detection and grading needs. Li et al. (2020) used hyperspectral techniques for deformed kiwifruit detection and compared three methods: the partial least-squares linear discriminant model, back-propagation neural network (BPNN), and least-squares support vector machine. The experimental results showed that the BPNN model achieved the highest accuracy at 97.56%. Fu et al. (2016) used a camera with a weight sensor on a grading line that was equipped for kiwifruit shape grading through a stepwise multiple linear regression method. The grading accuracy when using a linear combination of the cross-sectional diameter length was 98.3%. However, traditional image processing techniques, which generally extract feature targets manually, are only applicable to specific scene studies, have weaker robustness, and are susceptible to environmental influences during the extraction process.

Deep convolutional neural networks (CNNs) are superior to traditional methods and have been applied to the class classification and defect detection of fruits. Fan et al. (2020) improved the parameters and number of connections in a CNN model to detect the surface defects of apples in real time, with an accuracy of 92%. Lu et al. (2022) used the Attention-YOLOv4 model to detect the ripeness of different-colored apples. Zhang et al. (2020) improved the VGG16 model by converting it into a fully convolutional network and combining it with a spectral projection image to segment the mechanical damage and calyx regions of blueberries. Their method achieved an accuracy of 81.2%. Similarly, Wang et al. (2018) combined hyperspectral images with deep learning methods, and used the AlexNet and ResNet models to detect internal mechanical damage in blueberries. Their results showed that the deep learning models could maintain a higher accuracy than that of machine learning methods while reducing the calculation time significantly. Yu et al. (2018) proposed a combined model consisting of an autoencoder and a fully connected neural network to predict the hardness and soluble solid contents of Korla fragrant pears, resulting in a correlation coefficient of 0.89. Momeny et al. (2020) combined maximum pooling with mean pooling in a CNN to classify self-built regular and irregular cherry databases with an accuracy of 99.4%. Luna et al. (2019) created a dataset of healthy and defective tomatoes and evaluated the accuracy of their model using VGG16. A high accuracy rate of 98.75% was achieved. Azizah et al. (2017) used a four-fold cross-validation method to classify CNN mangosteen with an accuracy of 97.5%. Jahanbakhshi et al. (2020) proposed an improved CNN model for healthy and damaged sour lemon detection, achieving an accuracy of 100%. Xue et al. (2018) improved the YOLOv2 model using the Tiny-yolo-dense network to detect unripe mangoes with an accuracy of 97.02%. CNNs have achieved high detection accuracy, application flexibility, and good performance rates in many fruit quality detection studies. However, the detection of small objects with a low resolution remains challenging. This is because small objects with a low resolution provide few learning features and often coexist with larger undetectable objects.

Therefore, in this study, a kiwifruit dataset was constructed according to an image acquisition device based on grading lines for the detection of external kiwifruit defects. The widely used Yolov5s (Li et al., 2023) was selected as the base model. The network structure was improved and the loss function was optimized to achieve non-destructive and efficient external detection of kiwifruit. The results of this study can provide technical support for kiwifruit quality grading.

## 2 Materials and methods

### 2.1 Dataset production

#### 2.1.1 Sample source

**Figure 1:**
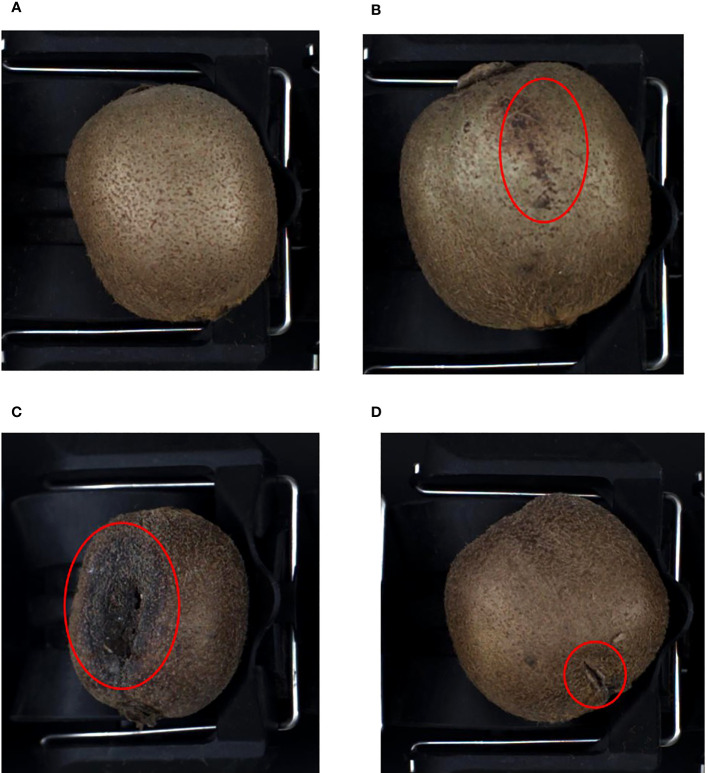
Kiwifruit samples. **(A)** Healthy, **(B)** leaf-rubbing damaged, **(C)** sunburned, **(D)** healed cuts or scarred.

Kiwifruit samples were obtained from the Zhouzhi (108.20 °E, 34.17 °N) and Meixian counties (107.76 °E, 34.29 °N) in Shaanxi Province. The kiwifruit varieties Xu Xiang and Cui Xiang were selected as the subjects of the study, and multiple batches were acquired in the field and online from November 2021 to November 2022. A total of 1,020 original samples were obtained, including 320 healthy samples, 240 leaf-rubbing damaged samples, 240 sunburned samples, and 220 healed cuts or scarred samples. The various sample types are presented in **Figure 1**.

#### 2.1.2 Image acquisition

**Figure 2:**
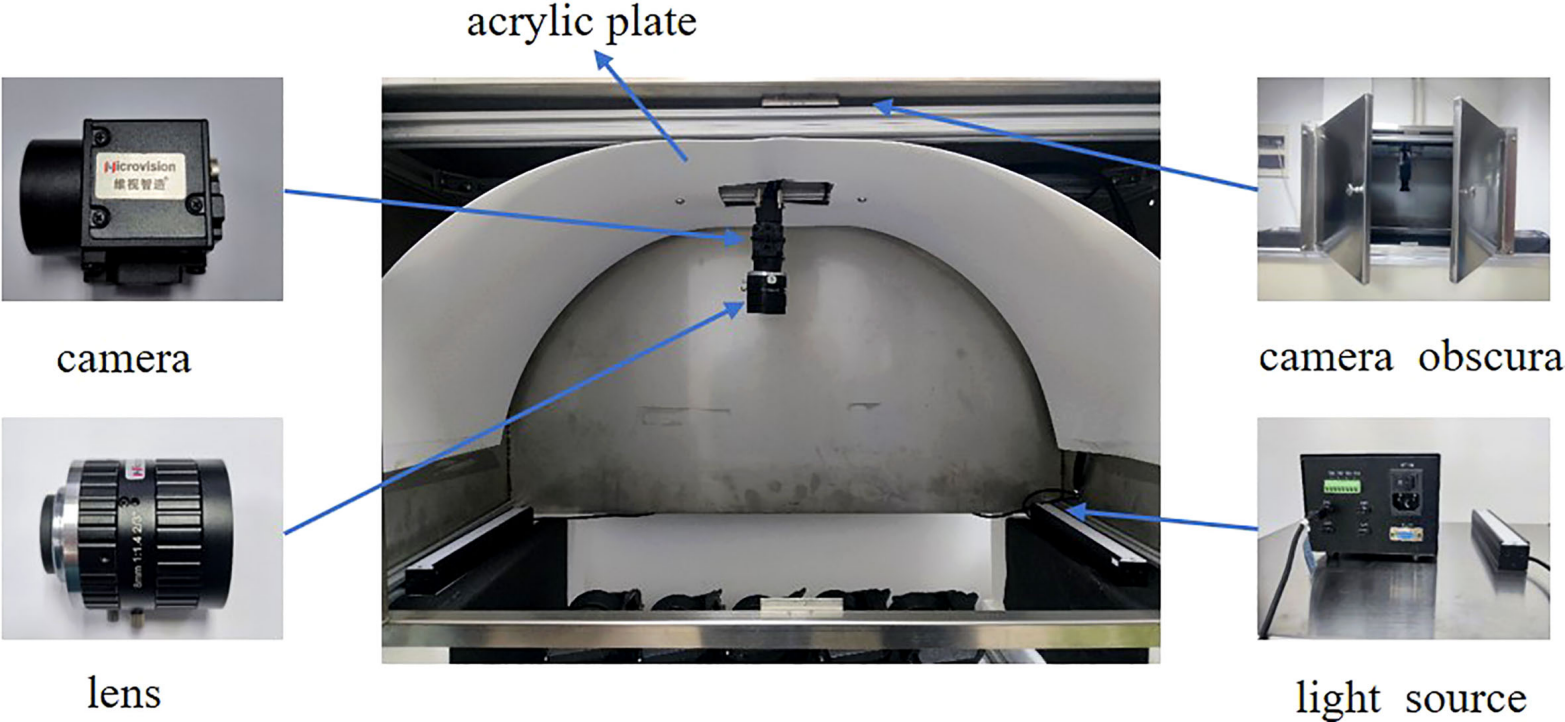
Acquisition device diagram.

**Figure 3:**
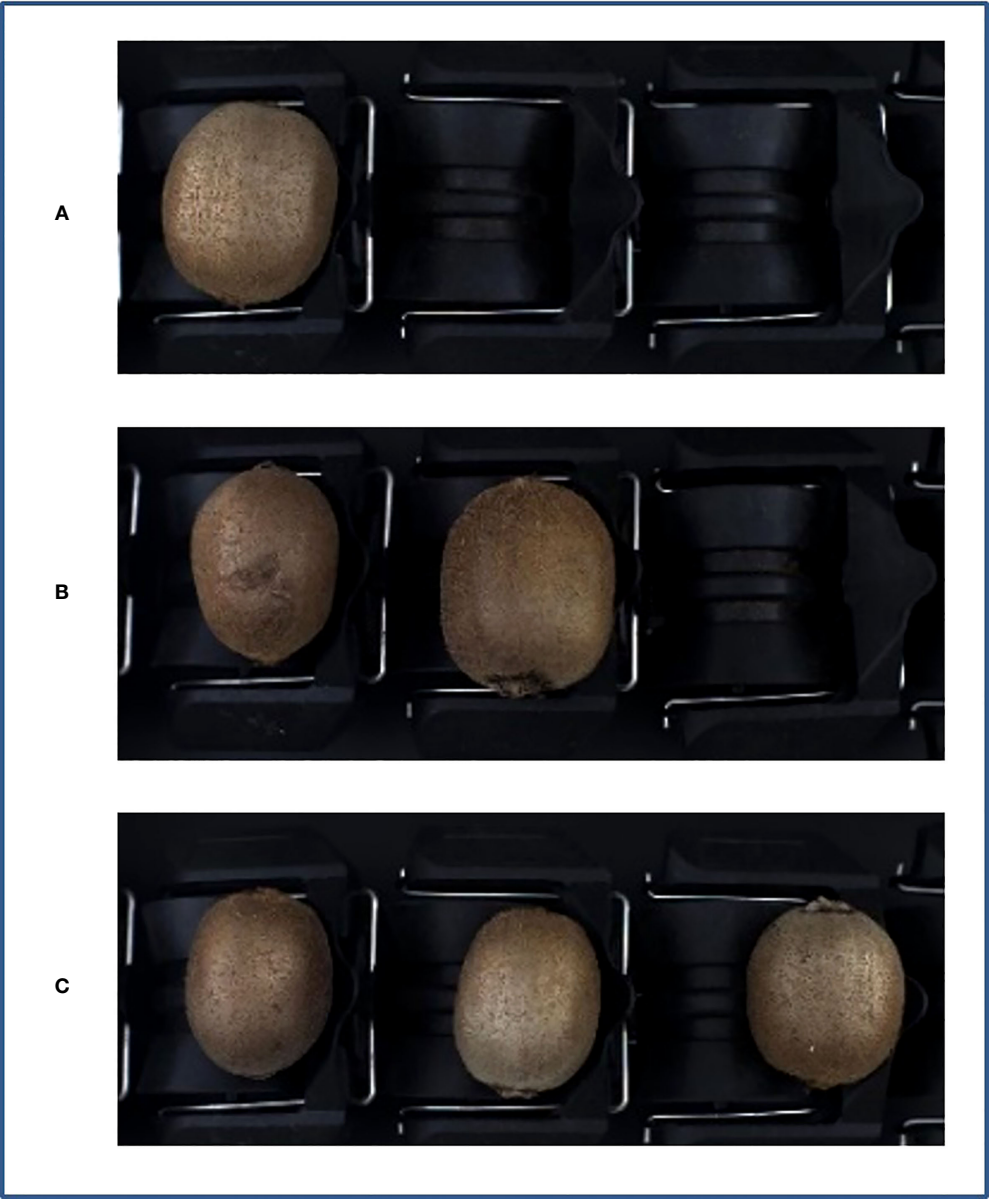
Image acquisition. **(A)** Single sample, **(B)** two samples, **(C)** three samples.

Image acquisition was performed using an MV-EM200C camera (Microvision, Xi’an, China) with a model BT-23C0814MP5 industrial lens, an image resolution of 1,600 × 1,200 pixels, and an acquisition frame rate of 39.93 fps. The image acquisition device was constructed based on a grading line (Li et al., 2018), as illustrated in **Figure 2**, and mainly included the camera, lens, camera obscura, light sources, and acrylic plate. The camera height was adjusted to 32 cm above the tray level to capture the information of the three trays completely in a single image for the grading application scenario. When the grading line moved, the roller tray could turn the kiwifruit, and three samples in a single image could be obtained to acquire the full surface information of the kiwifruit. The light source was emitted from the bottom and reflected on the kiwifruit surface through a half-cylinder acrylic plate, which helped to reduce the problems of uneven light exposure and reflection at different locations owing to direct radiation. When the graded line speed was adjusted to 3–5 pcs/sec, the pallet information was captured by a counter-light sensor, which was passed to the isolation plate, thereby driving the camera to trigger synchronously. Thus, the quality of the images captured by the device was improved. The captured images contained 1–3 unequal samples, with a total of 2,220 images captured, as shown in **Figure 3**.

#### 2.1.3 Data processing

First, the collected images were divided into training (1,332), validation (444), and test (444) sets by batch at a 3:1:1 ratio. A multi-data-enhanced fusion method based on an adjustable range was implemented to enhance the robustness of the model under background differences in the kiwifruit images. The training set data were randomly combined using six methods: contrast, brightness, and rotation angle adjustment, mirroring, Gaussian noise addition, and filtering. The training dataset was enhanced seven times, resulting in a total of 10,656 images. The specific parameters are listed in **Table 1**. The experiment was conducted using a dataset in the Pascal Voc format and the dataset was labeled using labelImg. Four categories were labeled: “Kiwifruit,” “Leaf-rubbing damaged,” “Sunburned,” and “Healed cuts or scarred,” with the latter three categories corresponding to each defect type. The sample labeled “Kiwifruit” was used to locate the kiwifruit, but a single sample labeled “Kiwifruit” was considered as healthy.

**Table 1:** Data enhancement methods.

Methods	Parameter range
Mirroring	/
Contrast ratio	(0.8, 1.2)
Gaussian noise	/
Filtering	/
Rotation angle	(-20°, 20°)
Brightness	(0.8, 1.2)

/, non-random variation.

### 2.2 Model construction

#### 2.2.1 Experimental environment

The experimental operating platform was a Dell Precision 7920 Tower workstation (Dell, Round Rock, TX, USA) with an Ubuntu 18.04 64-bit operating system. The central processor of the workstation was an Intel Xeon Silver 4216 @ 2.10 GHz (X2; Intel, Santa Clara, CA, USA) with 128 G of running memory. The GPU was an NVIDIA GeForce RTX 3090 (Nvidia, Santa Clara, CA, USA) with a 24 G display memory. A deep learning framework with a GPU was used to accelerate the dynamic neural network Pytorch version 1.11, Anaconda 3.7 environment manager, and Python version 3.8.

#### 2.2.2 Model structure

**Figure 4:**
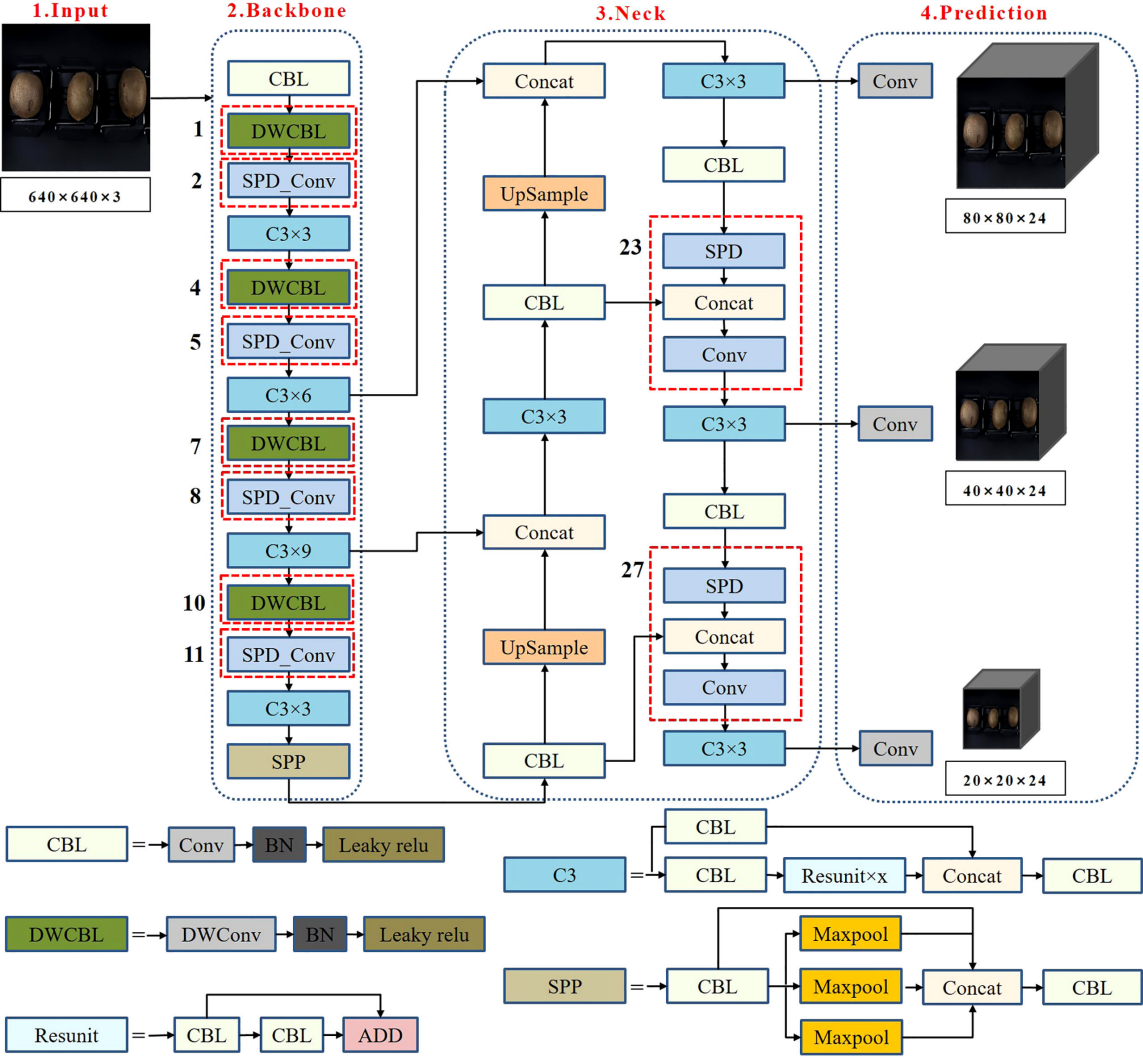
Yolov5-Ours network structure.

The structure of Yolov5-Ours, which was based on Yolov5s, is depicted in **Figure 4**. It included four parts: the input, backbone, neck, and prediction.

(a) Input: The input was a three-channel RGB image of kiwifruit, and the image size was uniformly adjusted from 1,600 × 1,200 to 640 × 640 at the acquisition time using adaptive picture scaling.

(b) Backbone: The backbone consisted of CBL, DWCBL, SPD-Conv, C3, and SPP. CBL consisted of convolutional and BN layers and leaky ReLU. The image size at the input was 640 × 640 × 3, and the output was 320 × 320 × 32 after slicing by the first CBL. DWCBL consisted of depth-wise separable convolution (DWConv) and BN layers and a Leaky ReLU. The DWConv layer with SPD-Conv (consisting of spatial-depth (SPD) and step-free convolutional layers) was implemented as the improved structure (the numbered part marked in **Figure 4**). The improved structure is described in detail in Section 2.3. C3 consisted of a CBL, residual structure, and convolutional layer connection, which could solve the problem of gradient repetition in the backbone network of the large CNN framework. Furthermore, it integrated the gradient changes into the feature map from beginning to end, thereby reducing the number of model parameters and computation values (Li et al., 2019) to ensure the speed and accuracy of the inference. SPP concatenated the different scales of the feature maps to expand the extraction of kiwifruit features using the maximum down-sampling of different convolutional kernels.

(c) Neck: FPN+PAN (Lin et al., 2017; Liu et al., 2018) was used. The FPN structure fuses and passes the feature information on the upper layers from top to bottom by up-sampling. The PAN structure is a bottom-up feature pyramid. The FPN+PAN structure was fused with feature layers from different backbone layers to improve the feature fusion capabilities further.

(d) Prediction: Output feature maps with sizes of 80 × 80, 40 × 40, and 20 × 20 were used to localize the kiwifruit defects. The training loss values were calculated using the loss calculation function and were iteratively updated to obtain the best model.

### 2.3 Structure optimization

#### 2.3.1 SPD-Conv module

The convolution and pooling layers that are used in conventional methods lead to the loss of fine-grained information and insufficient learned features in the image. This results in small and low-resolution kiwifruit defect features that cannot be learned effectively during the convolution process. To address this problem, we incorporated the convolutional structure of SPD-Conv (Sunkara and Luo, 2022) into Yolov5s instead of the convolutional and pooling layers. When the feature size of the kiwifruit was a feature mapping X with a size of M×M×C , to achieve a two-fold down-sampling operation, the scale value S was selected as 2 in Equation (1). Subsequently, the SPD layer was subjected to spatial sub-mapping $f_{0,0}、f_{0,1}、f_{1,0}、f_{1,1}$ by slicing. These spatial sub-mappings were spliced in the channel dimension to acquire the dimensional mapping $X'(\frac{M}{S=2},\frac{M}{S=2},4C)$ , and a step-free convolutional layer after SPD was added to obtain the final mapping $X''(\frac{M}{2},\frac{M}{2},C')$ . The SPD layer preserved the information in the channel dimension when down-sampling was performed in the feature layer by retaining all information in the channel dimension when down-sampling the feature layer. The step-free layer retained the feature discriminant information in the convolution and adjusted the number of output channels. As illustrated in **Figure 4**, SPD-Conv was used as a substitute for four convolutional layers with a step size of 2 to down-sample the feature map in the backbone. Similarly, two alternative operations were executed in the neck.


(1) $$\begin{array}{l} f_{0,0}=X[0:M:S,0:M:S],\cdots f_{0,\;S-1}=X[0:M:S,\;S-1:M:S]\\ f_{1,0}=X[1:M:S,0:M:S],\cdots f_{1,\;S-1}=X[1:M:S,\;S-1:M:S]\\ \vdots \\ f_{S-1,0}=X[S-1:M:S,0:M:S],\cdots f_{S-1,\;S-1}=X[S-1:M:S,\;S-1:M:S] \end{array}$$

#### 2.3.2 DWConv

**Figure 5:**
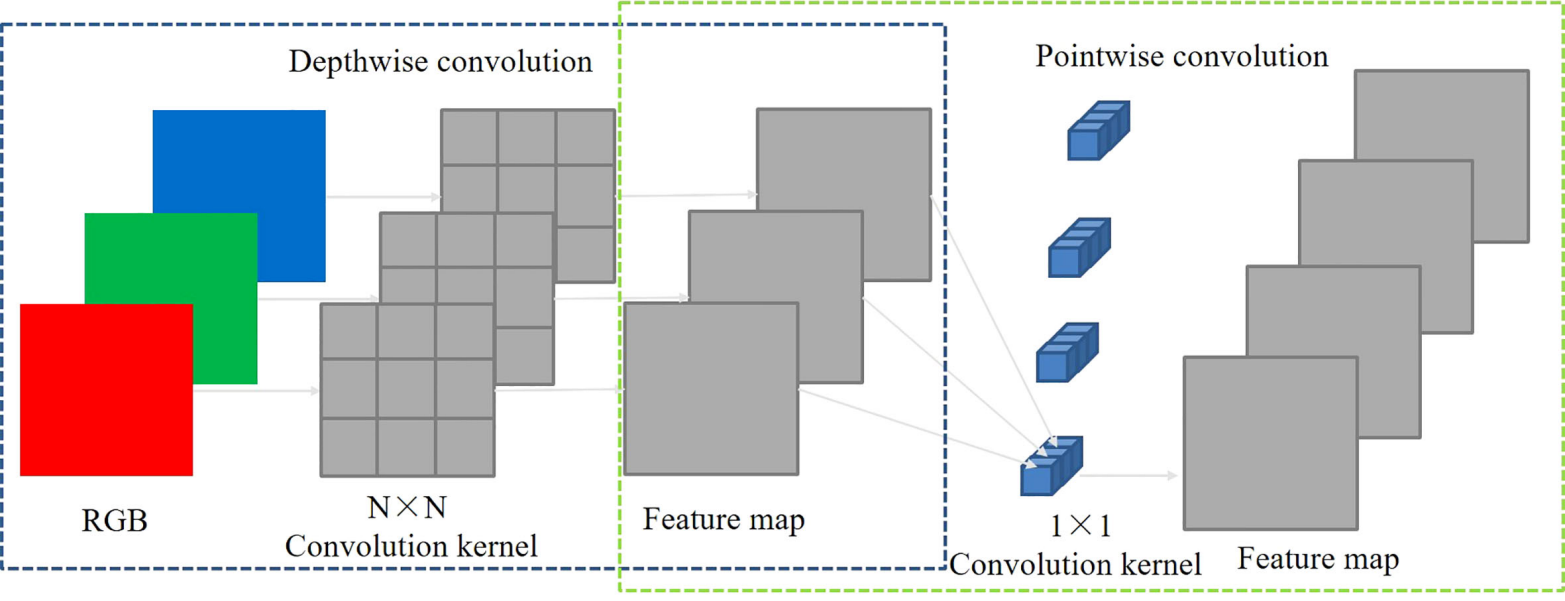
Schematic of DWConv.

The number of model calculation parameters and calculation amount increased following the structural improvement described in Section 2.3.1. We used DWConv (Chollet, 2017) instead of conventional convolution to solve this problem. The four regular convolutions in the backbone were replaced with DWConv, as indicated in **Figure 4**. As illustrated in **Figure 5**, the basic implementation process of DWConv consisted of depth-wise and point-wise convolution. Each convolution kernel of the depth-wise convolution convolved a single channel to make the number of input feature map channels the same as that of the output feature map channels. The point-wise convolution generated a new output feature map by linearly weighting the number of input feature map channels in the depth direction. DWConv effectively reduced the volume and computation of the parameters compared with conventional convolution for the same input and output cases.

### 2.4 Loss function

**Figure 6:**
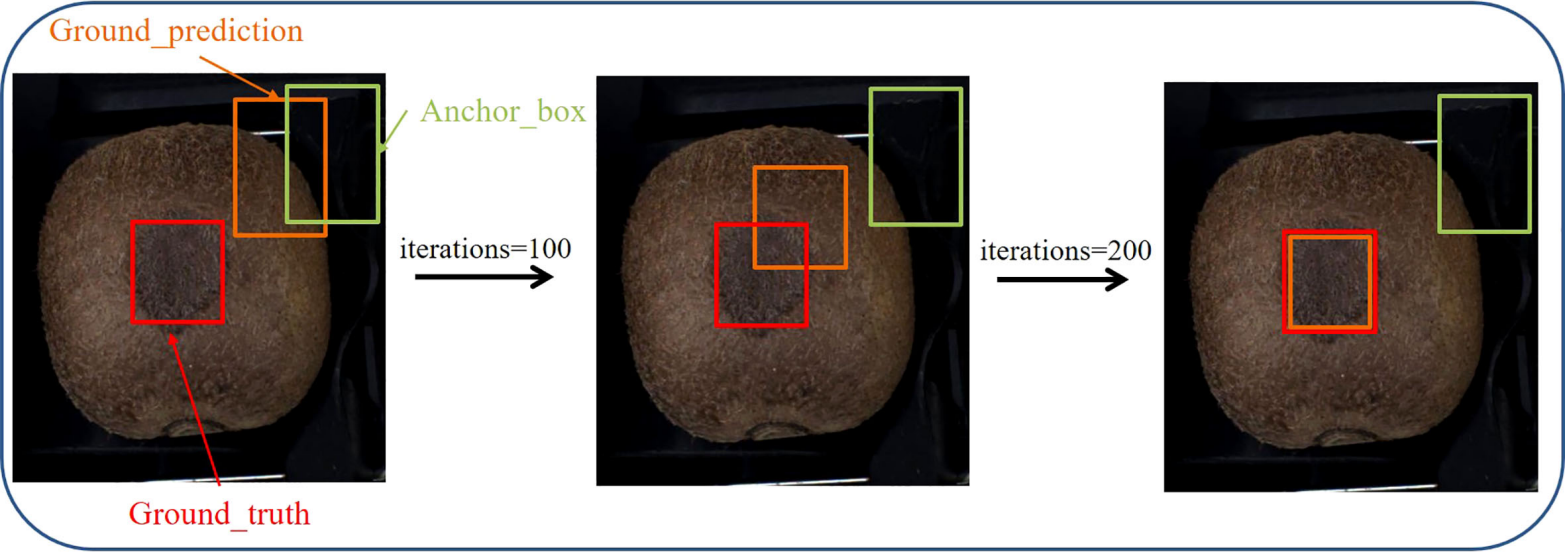
Schematic of EIOU implementation.


The target detection regression loss function IOU (Yu et al., 2016) cannot evaluate the distance information of the two frames when the prediction and target frames do not intersect. Thus, the gradient information cannot be passed back to the model, which results in the model not being learned and trained further. Moreover, when the prediction and target frames intersect, the model cannot reflect the overlapping method of both frames. GIOU (Rezatofighi et al., 2019) introduces the minimum outer rectangle concept into the prediction and target frames. Although it solves the problems of IOU, errors, difficult convergences, and horizontal and vertical instability occur when the prediction and target frames have inclusion relations. DIOU (Xu et al., 2023) improves the penalty term in GIOU to calculate the distance between the minimized center point of the prediction and target frames to accelerate the convergence. However, DIOU does not consider the aspect ratio in the regression process. CIOU adds the influence factor to the penalty term based on DIOU and considers the prediction frame aspect ratio as fitting the target frame aspect ratio. However, the aspect ratio that is described by CIOU is a relative value and may be ambiguous. EIOU (Zhang et al., 2022) replaces the aspect ratio with the width-height difference value based on CIOU and introduces the focal loss to solve the problem of imbalance between difficult and easy samples. Therefore, EIOU was used as the loss calculation function in this study. The implementation process is illustrated in **Figure 6** and the loss function value is calculated using Equation (2).

(2) $$\begin{array}{c} L_{EIOU}=L_{IOU}+L_{dis}+L_{asp}\\ =1-IOU+\frac{d^{2}(b^{P},b^{gt})}{{\left(w^{c}\right)}^{2}+{\left(h^{c}\right)}^{2}}+\frac{d^{2}(w^{P},w^{gt})}{{\left(w^{c}\right)}^{2}}+\frac{d^{2}(h^{P},h^{gt})}{{\left(h^{c}\right)}^{2}}, \end{array}$$

where $L_{IOU}$ is the overlap loss, $L_{dis}$ is the center distance loss, and $L_{asp}$ is the scale loss. Furthermore, $b^{P}$ and $b^{gt}$ are the coordinates of the center points of the prediction and target frames, respectively, whereas $d(b^{P},b^{gt})$ is the Euclidean distance between the frames. $w^{c}$ and $h^{c}$ are the width and height of the smallest outer rectangle of the prediction and target frames, respectively. Moreover, *IOU* is the ratio of the intersection of the prediction and target frames to the union, $d(w^{P},w^{gt})$ is the difference between the widths of the prediction and target frames, and $d(h^{P},h^{gt})$ is the difference between the lengths of the prediction and target frames.

### 2.5 Evaluation indicators

To evaluate the effectiveness of the external defect detection model for kiwifruit, multiple metrics were used, including the rate of precision and recall, number of parameters (Params) and FLOPs (Li et al., 2021), model size, average precision (AP) of a single sample, and average precision (mAP) of all categories. The precision and recall are determined by Equations (3) and (4), respectively.


(3) P=TP/(TP+FP)×100%

(4) R=TP/(TP+FN)×100%,

where P is the precision rate; that is, the proportion of predicted targets that are the same as the labeled targets, and R is the recall rate; that is, the proportion of correctly predicted positive samples to all labeled positive samples. TP represents the predicted positive and actual positive samples, FP represents the predicted positive and actual negative samples, and FN represents the predicted negative and actual positive samples.

The curve for PR was plotted with R and P as the horizontal and vertical coordinates, respectively, and the area enclosed by the curve was calculated to obtain AP . The calculation of mAP is shown in Equations (5) and (6).


(5) $$AP=\int_{0}^{1}P(R)\text{d}R\times 100\%$$

(6) $$mAP=\frac{1}{C}\sum_{c\in C}^{A}P(c)\times 100\%,$$

where c is a single category and C is all categories.

## 3 Results and discussion

### 3.1 Model training results

A stochastic gradient descent optimizer with a momentum of 0.937 and a weight decay of 0.0005 was selected to evaluate the performance of the proposed network. The number of training warm-up rounds, total number of rounds, and training batches were set to 3, 200, and 32, respectively. The training learning rate was set linearly from 0.003 to 0.01 following the warm-up phase and decayed linearly to a final value of 0.0001 after 200 iterations.

**Figure 7:**
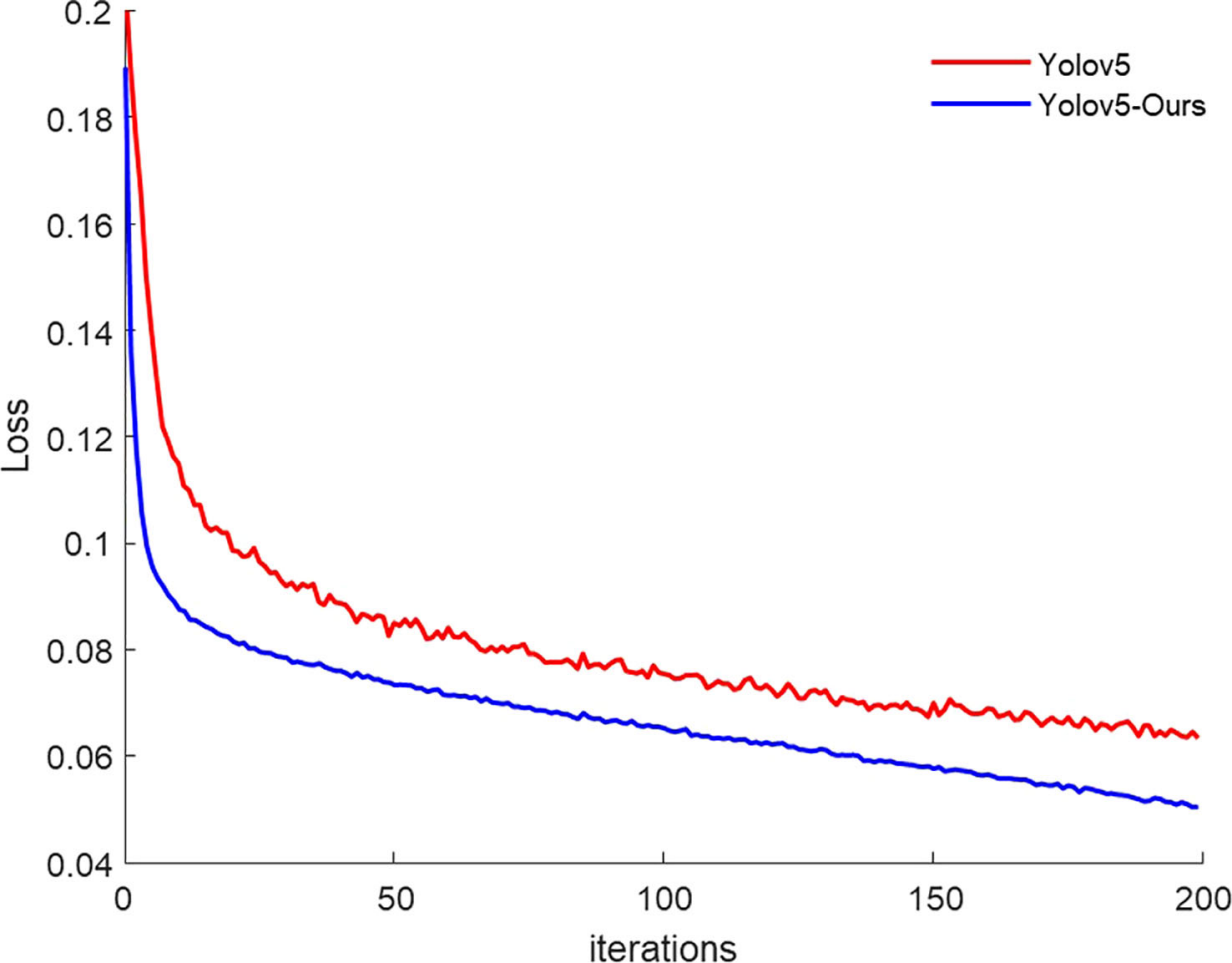
Training loss value.

The loss value is a metric that is used to measure the effectiveness of network training. **Figure 7** shows the loss values of Yolov5s and Yolov5-Ours in the training set. The loss value of Yolov5-Ours decreased rapidly to approximately 0.08 from the beginning of the iterations, and then steadily with an increase in iterations. The initial loss value of Yolov5s was larger than that of Yolov5-Ours; the loss value decreased more slowly and appeared to fluctuate with the increase in iterations. After 200 iterations, the loss value of Yolov5-Ours was 0.050 and that of Yolov5s was 0.063. Thus, Yolov5-Ours reduced the loss value by 0.013 compared to Yolov5s.

**Figure 8:**
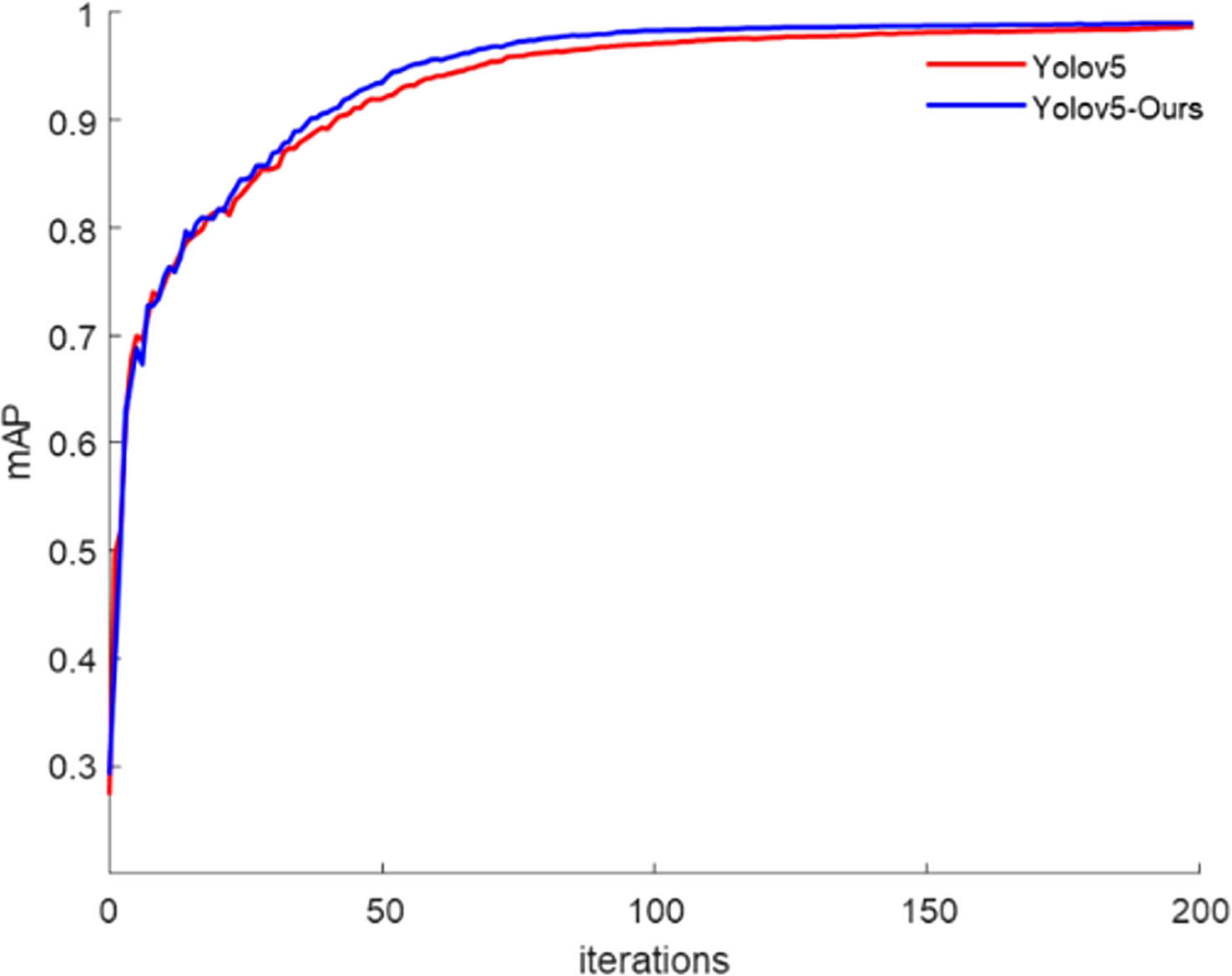
Average accuracy of training categories.

The AP of the training detection provides an important indication of whether the model has learned the features effectively. **Figure 8** depicts the average class detection accuracies of Yolov5s and Yolov5-Ours in the training set. From the beginning of the iterations, the detection mAP increased while Yolov5s and Yolov5-Ours learned the kiwifruit defect features. Yolov5-Ours reached convergence at 100 iterations and the detection mAP was slightly higher than that of Yolov5s. After 200 iteration rounds, both Yolov5s and Yolov5-Ours reached stability, and both had better detection mAPs for kiwifruit defects, but that of Yolov5-Ours was slightly higher than that of Yolov5s. The Yolov5-Ours model achieved a detection accuracy of 99.4% for healthy kiwifruit, 99.3% for leaf-rubbing damaged kiwifruit, 97.7% for healed cuts or scarred kiwifruit, and 99.2% for sunburned kiwifruit during the validation phase on 444 kiwifruit images.

The number of parameters and computations were visualized in terms of the spatial and temporal complexity for the model size and speed, respectively. Spatial complexity refers to the consumption of computer hardware memory resources, whereas temporal complexity is the model computation time. The number of parameters and amount of computation during the training process of Yolov5s, Yolov5s+SPD-Conv, and Yolov5-Ours were determined, as indicated in **Table 2**. The number of parameters of Yolov5s+SPD-Conv increased by 1.54 M and the computation amount increased by 17.5 G compared to Yolov5s. The number of parameters of Yolov5-Ours decreased by 1.56 M and the computation amount decreased by 15.1 G compared to Yolov5s+SPD-Conv. These results demonstrate the effectiveness of the model improvement described in Section 2.3.1.

**Table 2:** Number of parameters and calculated values.

Model	Params (M)	FLOPs (G)
Yolov5s	7.03	15.9
Yolov5s+SPD-Conv	8.57	33.4
Yolov5-Ours	7.01	18.3

### 3.2 Model testing results

**Figure 9:**
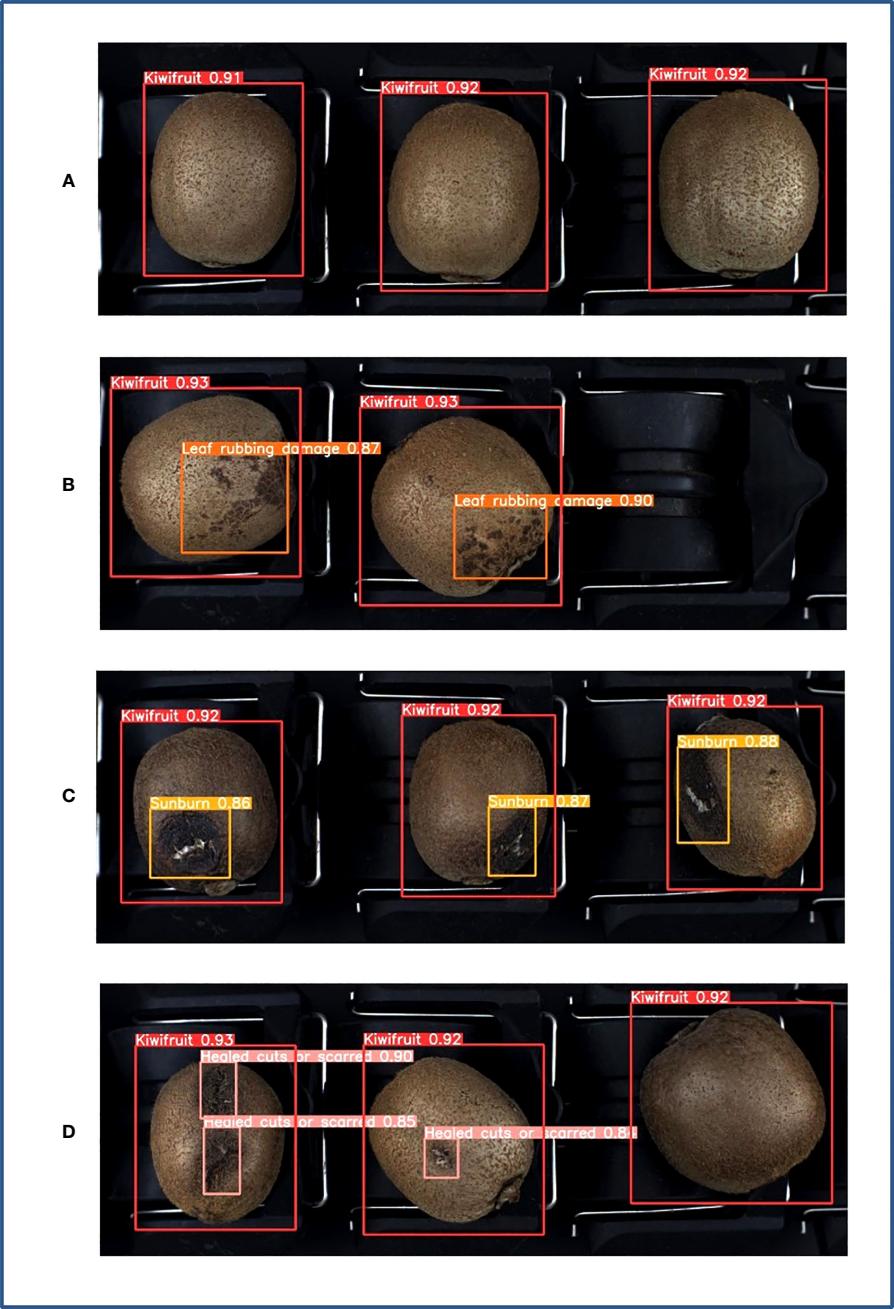
Test results. **(A)** Healthy, **(B)** leaf-rubbing damaged, **(C)** sunburned, **(D)** healed cuts or scarred.

The 444 test set images contained 1,151 kiwifruit samples, including 326 healthy, 268 leaf-rubbing damaged, 284 healed cuts or scarred, and 273 sunburned samples. The samples in each category were tested using Yolov5-Ours with optimal weights. As indicated in **Table 3**, the precision rates for the four categories were all higher than 99% and the recall rates were all higher than 95%. The average detection precisions of the healthy, leaf-rubbing damaged, healed cuts or scarred, and sunburned samples were 98.8%, 98.7%, 97.6%, and 95.9%, respectively, at a confidence threshold of 0.5, whereas the detection mAP of all categories was 97.7%. Moreover, the detection time of the image was only 8.0 ms, thereby meeting the real-time sorting requirements of the grading line. As shown in a partial plot of the results (**Figure 9**), Yolov5-Ours could effectively detect all categories at a confidence level higher than 0.8 for each category, which suggests that the model is highly adaptable and robust for each category of kiwifruit.

**Table 3:** Test results.

Category	P (%)	R (%)	AP@0.5 (%)	mAP@0.5 (%)	Image (ms)
Healthy	99.8	97.1	98.8	97.7	8.0
Leaf-rubbing damaged	99.7	96.7	98.7		
Healed cuts or scarred	99.5	98.3	97.6		
Sunburned	1.0	95.1	95.9		

### 3.3 Model comparison

The sample mAP and model sizes of SSD, Yolov5s, Yolov7, and Yolov5-Ours were compared to validate the performance of Yolov5-Ours further. As shown in **Table 4**, the mAP of the samples was compared at confidence threshold values of 0.5 and 0.8. When the confidence level was 0.5, the mAP of Yolov5-Ours was 1.1% lower than that of Yolov7, but 10% and 6.4% higher than those of SSD and Yolov5s, respectively. When the confidence level was 0.8, the mAP of Yolov5-Ours was 88.3%, 15.5%, and 10.1% higher than those of SSD and Yolov5s, but 3.2% lower than that of Yolov7. The model size of Yolov5-Ours was the same as that of Yolov5s, which was approximately 6.5- and 4-fold smaller than those of SSD and Yolov7, respectively.

**Table 4:** Model comparison.

Model	mAP@0.5 (%)	mAP@0.8 (%)	Weight (MB)
SSD	87.7	72.8	108.1
Yolov5s	91.3	78.2	14.4
Yolov7	98.8	91.5	74.8
Yolov5-Ours	97.7	88.3	14.4

SSD is mainly divided into the backbone network and multi-scale prediction network. The backbone network adopts the VGG16 model, which is used to realize the initial extraction of image features. The multi-scale feature detection network extracts the feature layers that are obtained from the backbone network at different scales, so that different feature maps can detect different-sized features. Finally, the detection results are regressed. Yolov7 introduces model reparameterization into the network structure, includes a new label assignment method, and incorporates multiple tricks for efficient training compared to Yolov5. Yolov7 achieves higher computational efficiency and accuracy than Yolov5, and can achieve better detection accuracy with the same computational resources. However, Yolov5 is much faster than Yolov7 in terms of the inference speed, because the faster computational efficiency of Yolov7 leads to more memory-occupied resources. Yolov5-Ours improves the detection of small feature defects on the surface of kiwifruit by adding the SPD-Conv module based on Yolov5s and reduces the parameters using DWConv, which means that the model size does not increase even with higher detection accuracy. In summary, the results verified that Yolov5-Ours balances the model size and accuracy and achieves efficient performance in kiwifruit defect detection.

## 4 Conclusions

We developed and validated the effectiveness of a non-destructive detection method for kiwifruit defects. We applied the target detection technique to multiple healthy and defective kiwifruits and improved several aspects, including the data acquisition and methodology, to detect kiwifruit defects in various categories efficiently. First, a kiwifruit image acquisition device was constructed and improved to solve the problem of uneven light exposure in the image, thereby improving the image quality. Subsequently, a kiwifruit database was established. To avoid the problem of overfitting, the training dataset was increased seven-fold using a new data enhancement method. We proposed Yolov5-Ours based on Yolov5s, in which we fused SPD-Conv and DWConv and improved the loss calculation function. The average detection accuracy of healthy, leaf-rubbing damaged, healed cuts or scarred and sunburned samples was 97.7%. The single-frame image detection was run in 8.0 ms, thereby meeting the classification line-sorting requirements. The results validated the effectiveness of Yolov5-Ours in terms of both the accuracy and model size.

The external kiwifruit defects of sunburned and healed cuts or scarred affect the flesh of the kiwifruit, and effective detection can increase the commercial value of the kiwifruit. Leaf-rubbing damaged kiwifruit only has defects in the skin and the flesh of the kiwifruit is normal, and correct detection can increase the reuse of iso-extracted fruits. Consequently, the proposed method can facilitate the effective detection of kiwifruit defects, provide a theoretical basis for online real-time detection and grading, and serve as a framework for future non-destructive defect detection in agricultural products.

This study also has some shortcomings. Only three major kiwifruit defects were selected for detection and sorting. We plan to expand the categories of kiwifruit defects for detection in the future, which will make the study more applicable to actual kiwifruit sorting.

## Data availability statement

The raw data supporting the conclusions of this article will be made available by the authors, without undue reservation.

## Author contributions

FW and BZ designed the study, performed the experiments, analyzed the data, and wrote the manuscript. CL supervised the project and helped to design the research. LD, XL, and PG performed the experiments. All authors have contributed to the manuscript and approved the submitted version.
